# Artemether-Lumefantrine Pharmacokinetics and Clinical Response Are Minimally Altered in Pregnant Ugandan Women Treated for Uncomplicated Falciparum Malaria

**DOI:** 10.1128/AAC.01605-15

**Published:** 2016-02-26

**Authors:** Myaing M. Nyunt, Vy K. Nguyen, Richard Kajubi, Liusheng Huang, Joshua Ssebuliba, Sylvia Kiconco, Moses W. Mwima, Jane Achan, Francesca Aweeka, Sunil Parikh, Norah Mwebaza

**Affiliations:** a Institute for Global Health, University of Maryland Baltimore School of Medicine, Baltimore, Maryland, USA; b Department of Clinical Pharmacy, University of California, San Francisco, San Francisco, California, USA; c Infectious Disease Research Collaboration, Makerere University, Kampala, Uganda; d Yale School of Public Health, New Haven, Connecticut, USA

## Abstract

Artemether-lumefantrine is a first-line regimen for the treatment of uncomplicated malaria during the second and third trimesters of pregnancy. Previous studies have reported changes in the pharmacokinetics and clinical outcomes following treatment with artemether-lumefantrine in pregnant women compared to nonpregnant adults; however, the results are inconclusive. We conducted a study in rural Uganda to compare the pharmacokinetics of artemether-lumefantrine and the treatment responses between 30 pregnant women and 30 nonpregnant adults with uncomplicated Plasmodium falciparum malaria. All participants were uninfected with HIV, treated with a six-dose regimen of artemether-lumefantrine, and monitored clinically for 42 days. The pharmacokinetics of artemether, its metabolite dihydroartemisinin, and lumefantrine were evaluated for 21 days following treatment. We found no significant differences in the overall pharmacokinetics of artemether, dihydroartemisinin, or lumefantrine in a direct comparison of pregnant women to nonpregnant adults, except for a statistically significant but small difference in the terminal elimination half-lives of both dihydroartemisinin and lumefantrine. There were seven PCR-confirmed reinfections (5 pregnant and 2 nonpregnant participants). The observation of a shorter terminal half-life for lumefantrine may have contributed to a higher frequency of reinfection or a shorter posttreatment prophylactic period in pregnant women than in nonpregnant adults. While the comparable overall pharmacokinetic exposure is reassuring, studies are needed to further optimize antimalarial efficacy in pregnant women, particularly in high-transmission settings and because of emerging drug resistance. (This study is registered at ClinicalTrials.gov under registration no. NCT01717885.)

## INTRODUCTION

Malaria, the most important global parasitic disease, carries a high burden of morbidity and mortality, particularly in children and pregnant women residing in sub-Saharan Africa (1–3), where malaria exposure occurs in an estimated 12.4 million pregnancies annually (4). Malaria parasitemia during pregnancy, with or without clinically symptomatic illness, places women at a higher risk of severe maternal anemia (5). Placental sequestration of Plasmodium falciparum parasites (6, 7) is strongly associated with adverse pregnancy outcomes, including intrauterine growth restriction and/or prematurity, resulting in low birth weight of the newborn or spontaneous abortion (8–11).

A fixed-dose oral formulation of artemether plus lumefantrine (AL), an artemisinin-based combination therapy (ACT), is a first-line regimen for uncomplicated P. falciparum malaria in pregnant women in the second or third trimester (12). Artemether is rapidly converted to its active metabolite dihydroartemisinin (DHA) (13, 14). Both compounds have potent antimalarial activity and are responsible for rapid reductions in parasite biomass (15–17). Lumefantrine is absorbed more slowly, which can be impacted by multiple factors, including food intake (18), and it has a longer active terminal half-life, which helps to eradicate residual parasites and is critical in protecting the host against recurrent infection (19, 20).

Both artemether and lumefantrine undergo cytochrome P450 (CYP) metabolism (14), with final systemic clearance via uridine glucuronosyltransferase (UGT) enzymes (21, 22), both of which may be altered by malaria infection (23, 24) and pregnancy-related physiologic changes (25). The induction of these pathways may lead to a reduction in AL pharmacokinetic (PK) exposure during pregnancy (26, 27), thereby increasing the risk of reinfection, treatment failure (recrudescence), or selective pressures for the development of drug resistance (20, 28, 29). However, drug dosing strategies for pregnant women are typically not guided by these and other physiologic changes in pregnancy and are the same as those for nonpregnant adults (30, 31). AL PK has been studied in pregnant women from Thailand, Uganda, and Tanzania (26, 27, 32–37). These studies reported various magnitudes of PK changes, but all suggested that artemether, DHA, and lumefantrine PK parameters may be reduced in pregnant women compared to those in nonpregnant adults. The impact of this reduction in PK exposure on clinical outcomes for AL is unclear; a study in Thailand suggested low concentrations were associated with suboptimal cure rates, while the high overall cure rates in Africa precluded the ability to test for PK-recrudescence associations. While the potential determinants of malaria treatment outcomes are many, it is largely agreed that lumefantrine exposure is a critical determinant (19, 38, 39). It has been suggested that lumefantrine concentration, often cited as 175 ng/ml or 280 ng/ml, should be kept above a certain threshold on day 7 to minimize the risk of treatment failure (34, 38, 40). However, more definitive exposure-response studies are needed in pregnant women in sub-Saharan Africa, where malaria transmission patterns and intensities differ. To fill this knowledge gap, we conducted a prospective intensive PK clinical study (registered at ClinicalTrials.gov under registration no. NCT01717885) with extended PK sampling and follow-up to directly compare artemether, DHA, and lumefantrine PK and treatment outcomes between HIV-uninfected pregnant women and nonpregnant adults in Tororo, Uganda, where P. falciparum transmission is high and holoendemic (41).

## MATERIALS AND METHODS

### Study design.

A prospective single-center open-label clinical PK cohort study was conducted to compare AL PK and treatment outcomes between the pregnant and nonpregnant adults treated for uncomplicated P. falciparum malaria. The inclusion criteria included a diagnosis of uncomplicated malaria (defined as a fever [tympanic temperature of ≥38°C or history of fever within the past 24 h) and microscopy-confirmed P. falciparum monoinfection), age ≥16 years, and a confirmed pregnancy of 12 to 38 weeks gestational age. The exclusion criteria included severe malaria, as defined by the World Health Organization (WHO) (42), or significant other illnesses (such as HIV and tuberculosis [TB]), hemoglobin (Hb) level of <7.0 g/dl, concurrent use of medications with potential interactions with the study drugs, and antimalarial drug treatment within 2 weeks prior to study enrollment. HIV-negative status was confirmed by two rapid diagnostic tests. The study was independently approved by the Uganda National Council for Science and Technology (Kampala, Uganda), the Makerere University School of Medicine Research and Ethics Committee (Kampala, Uganda), the Yale University Human Investigations Committee (New Haven, CT), and the University of California, San Francisco Committee on Human Research (San Francisco, CA).

### Clinical procedures.

**FIG 1:**
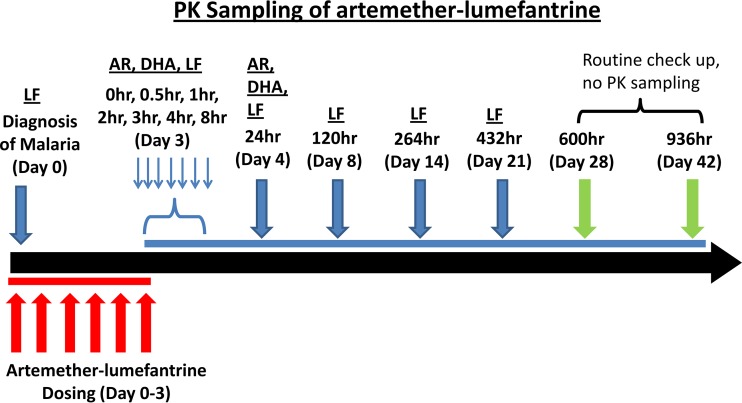
Blood for pharmacokinetic analyses was collected on study day 0 (prior to first dose) and before and 0.5, 1, 2, 3, 4, 24, 120, 264 h (day 14), and 432 (day 21) h after the last (6th dose). While the 120-h time point technically fell on day 8 in this study, the 120-h time is consistent with day 7 values previously reported in the literature, since in both cases, this time point is 120 h after the last AL dose. Thus, for our study, the 120-h/day 8 time point is referred to as day 7 throughout. AR, artemether; LF, lumefantrine.

Participants were recruited from the Tororo District Hospital and referral clinics from January 2013 to February 2014. Informed consent was obtained in each participant's local language, as appropriate. For women, pregnancy was confirmed by a urine test, and gestational age was determined by the last menstrual period, clinical examination, and ultrasound. All participants were provided an insecticide-treated bed net at time of enrollment, and all pregnant women received two doses of sulfadoxine/pyrimethamine at 16 to 24 weeks and 28 to 36 weeks gestation, in accordance with Ugandan guidelines. Day 0 was designated the first day of AL treatment. Six doses of 80 mg of artemether and 480 mg of lumefantrine (four tablets of Coartem; Novartis Pharma AG, Basel, Switzerland), with 200 ml of milk to provide adequate fat to enhance and control for lumefantrine absorption (18), were administered over a dosing schedule adjusted to permit the timing of the 6th dose to occur in the morning of day 3 to allow intensive PK blood collection during the day. Dosing was performed so as to ensure that the first 2 doses were administered on the day of diagnosis, followed by dosing intervals of no more than 16 h for the doses 3 and 4. Doses 5 and 6 were 12 h apart. A full dose was repeated if vomiting occurred within 30 min. The enrolled participants were clinically evaluated in the study clinic with active and passive surveillance on study days 0, 1, 2, 3, 4, 8, 14, 21, 28, and 42 (Fig. 1). Participants were given study contact information for emergencies and encouraged to come to the clinic (open 7 days a week) anytime they felt unwell.

The scheme of blood collection for various laboratory analyses is displayed in Fig. 1. At each visit, blood was collected for thin and thick malaria blood smears and on filter papers for parasite genotyping. Complete blood count and liver function tests were performed on study days 0, 14, and 28. Blood was collected for lumefantrine PK analysis on day 8 instead of day 7, which is typically reported in the published literature, since AL dosing in our study was extended to day 3 instead of day 2 to allow for intensive blood sampling to occur during the day. Thus, we refer to day 8 results here as “day 7” to permit a convenient comparison to prior studies. Blood for the PK analyses was collected (Fig. 1) on study day 0 (prior to first dose), before and after the last (6th) dose on day 3 at 0, 0.5, 1, 2, 3, 4, 8, and 24 h, and on study days 7, 14, and 21. Both capillary and venous blood were simultaneously collected at 2 and 24 h after the last dose and on day 7 to assess the correlation between the capillary and venous plasma lumefantrine concentrations (37, 43), and only capillary blood was collected at later time points. Only participants who completed the full dosing regimen were included in the intensive PK study. For PK analyses, 200 to 500 μl of blood was collected in a K_3_EDTA-coated tube, immediately placed on ice, and centrifuged at 800 × *g* for 10 min at 4°C. Plasma was separated, split into aliquots, and stored at −70°C until analysis.

### Laboratory methods.

Giemsa-stained thick and thin blood smears were evaluated to quantify parasite density and identify isolates to the species level, respectively. Parasite densities were calculated by counting the number of asexual parasites per 200 leukocytes, assuming a leukocyte count of 8,000/μl. A blood smear was declared negative when no asexual parasites were seen under 100 high-power fields. Dried blood spots collected on filter paper were used to distinguish recrudescent and new infections for all recurrent episodes of malaria, using previously described methods (44). DNA was isolated from blood spots, and samples were genotyped in a stepwise fashion using 6 polymorphic markers (merozoite surface protein [MSP]-1, MSP-2, and MSP-4 microsatellites). If, for any of the 6 loci, an allele was not shared between consecutive episodes of parasitemia, the episode was classified as a new infection. If at least 1 allele was shared at all 6 loci, the episode was classified as recrudescent.

### Assay analysis of artemether, DHA, and lumefantrine.

Plasma concentrations of the study drugs were determined using high-performance liquid chromatography-tandem mass spectrometry (LC-MS/MS), as previously described (45, 46). Artemether and DHA concentrations were quantified from 50-μl plasma samples, with the calibration range between 0.5 and 200 ng/ml and a lower limit of quantification (LLOQ) of 0.5 ng/ml for both artemether and DHA (46). The % coefficient of variation (%CV) for quality control (QC) samples was <10% for artemether and DHA. Lumefantrine concentration was quantified from 25-μl plasma samples using an LC-MS/MS method, with a calibration range of 50 to 20,000 ng/ml, and the LLOQ was set to 50 ng/ml, as previously described (45). The %CV during analysis for lumefantrine was <5%.

### Data analysis.

The primary endpoints were the PK parameters of artemether, DHA, and lumefantrine. These included the area under the plasma concentration-time curve from 0 to 8 h and 0 to 24 h (AUC_0–8_ and AUC_0–24_, respectively, for artemether and DHA, and AUC_0–∞_ for lumefantrine), maximal concentration (*C*_max_), time to *C*_max_ (*T*_max_), terminal elimination half-life (*t*_1/2_), and plasma concentrations of lumefantrine on days 7 (*C*_7_), 14 (*C*_14_), and 21 (*C*_21_). Secondary endpoints included the type, severity, and frequency of adverse events (AEs) using the grading criteria developed and used by the Division of AIDS, NIAID, to report AEs in AIDS clinical trials (47), and PCR-confirmed malaria recrudescence or new infection at 28 and 42 days of follow-up, using the WHO criteria, defined as adequate clinical and parasitological response (ACPR), early treatment failure, late clinical failure (LCF), and late parasitological failure (LPF) (48).

Noncompartmental analysis of plasma drug concentrations was performed using WinNonlin (version 6.30; Certara L.P., Pharsight Corporation, Mountain View, USA). Plasma samples below the LLOQ were treated as missing data except for (i) the predose concentration, which was set to 0 if below the LLOQ, and (ii) when the first time point below the LLOQ after *C*_max_ was necessary to adequately estimate an AUC for artemether or DHA, at which time 0.5 LLOQ was assigned. A linear-up/log-down trapezoidal method with first-order input was used to calculate total exposure, defined as the AUC from time 0 to 8 h (AUC_0–8_) and 0 to 24 h (AUC_0–24_). The extrapolated AUC from 0 h to infinity (AUC_0–∞_) was determined by dividing the last measured concentration by the terminal elimination rate constant (λ_z_). Extrapolation to infinity was carried out only if there were at least 3 measurable concentrations following the peak concentration (including peak point). The elimination rate constant (λ_z_) was estimated using the program's best fit feature combined with manual fine tuning in some cases. The *t*_1/2_ was calculated as ln(2)/λ_z_. The maximum concentration (*C*_max)_ and time to maximum concentration (*T*_max_) for lumefantrine and concentrations at 24 h (*C*_1_) and on days 7 (*C*_7_) and 14 (*C*_14_) were reported as observed.

The data were analyzed using Stata version 12.1 (StataCorp, College Station, TX, USA). A sample size of 30 in each group was targeted to detect a 35% difference in the AUC of all analytes with 80% power and 5% significance level. PK parameters were compared between the two study groups using a Wilcoxon rank sum or chi-square test, as appropriate.

## RESULTS

### Study profile.

A total of 104 patients presented with suspected malaria; 72 patients were eligible, consented, and enrolled, and 61 patients (31 pregnant women and 30 nonpregnant adults) completed the study. One pregnant woman delivered on day 11 and was excluded, leaving 60 adults (30 pregnant and 30 nonpregnant adults) in the final data analysis. Reasons for withdrawal (*n* = 11) were elective discontinuation for reasons deemed not related to the study or study drugs (*n* = 5), incorrect drug dosing (*n* = 2), missed clinic visits (*n* = 2), early delivery (*n* = 1), and error in the determination of pregnancy status for a woman initially assigned to the nonpregnant group (*n* = 1). Table 1 displays the baseline characteristics among pregnant and nonpregnant adults. The pregnant women were divided nearly equally between 2nd and 3rd trimesters at the time of PK evaluations. The body weight and body mass index (BMI) were significantly higher in the pregnant group (*P* < 0.05), as expected. Notably, out of the 30 nonpregnant adults, 28 (93%) adults were underweight, according to the WHO BMI classification (49). The parasite density at the time of diagnosis was significantly higher in pregnant women than in nonpregnant adults (*P* < 0.001), with geometric means (95% confidence interval) of 13,227 parasites/μl (7,728 to 22,639 parasites/μl) and 597 parasites/μl (261 to 1,371 parasites/μl), respectively. Hemoglobin was significantly lower in pregnant versus nonpregnant adults, with 9 of 30 pregnant women (30%) and none of the nonpregnant adults having moderate to severe anemia (hemoglobin, <10 g/dl). Finally, 5 pregnant women and 2 nonpregnant adults had detectable gametocytes at the time of diagnosis, and 23% of pregnant women and 10% of nonpregnant adults had detectable gametocytes at some point during follow-up.

**TABLE 1:** Baseline characteristics of study participants

Characteristic^*a*^	Pregnant women (*n* = 30)^*b*^	Nonpregnant adults (*n* = 30)
Age (yr)	25 (18–39)	24 (16–68)
% female	NA	63
Body wt (kg)	59.4 (44.5–81.1)	55.7 (38.0–68.4)
BMI	21.9 (17.4–28.9)	20.4 (14.3–44.8)
Gestational age (wk)	28 (14–34)	NA
No. (%) in 2nd trimester	14 (47)	NA
Parasite density (geometric mean [95% CI]) (parasites/μl)	13,227 (7,728–22,639)	597 (261–1,371)
White blood cell count (10^3^/ml)	6.0 (2.4–8.5)	4.8 (2.7–9.5)
Neutrophil count (10^3^/ml)	4.0 (1.3–6.4)	2.0 (1.1–7.4)^*c*^
Platelet count (10^3^/ml)	142 (36–309)	167 (30–288)
Hemoglobin level (g/dl)	10.5 (7.6–13.1)	13.3 (11.8–17.1)
No. (%) with hemoglobin <10 g/dl^*d*^	9 (30)	0
Alanine aminotransferase (IU)	12 (7–43)	19 (11–63)
Aspartate aminotransferase (IU)	23 (12–57)	28.5 (18–73)
Serum creatinine (mg/ml)	0.64 (0.17–1.27)	0.99 (0.46–1.53)

a All values are expressed as the median (range), unless otherwise noted. CI, confidence interval.

b Does not include the one pregnant subject who delivered on day 11. NA, not applicable.

c *n* = 29; one patient had undetectable neutrophils due to assay issues and was normal upon rechecking.

d Moderate-severe anemia, as per WHO definition (42).

### Pharmacokinetic parameters.

**FIG 2:**
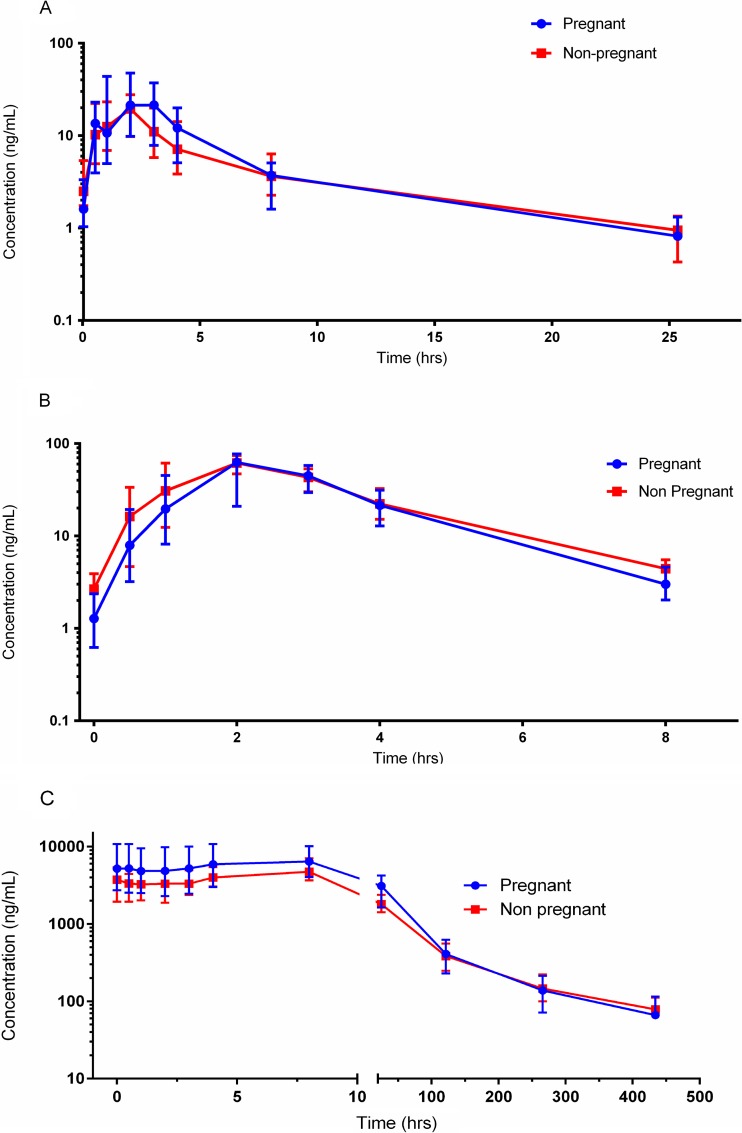
Time-plasma concentration plot of artemether (A), dihydroartemisinin (B), and lumefantrine (C) in pregnant women and nonpregnant adults with uncomplicated malaria. The median concentrations are reported, with the error bars indicating interquartile ranges.

The PK parameters are summarized in Table 2. Overall, there were no significant differences in the AUCs or *C*_max_ for artemether, DHA, or lumefantrine between pregnant and nonpregnant adults (Table 2 and Fig. 2). The lumefantrine concentrations on days 7 and 14 were not significantly different between the two study groups, but the estimated terminal half-lives for DHA and lumefantrine (Table 2 and Fig. 2) were significantly shorter in pregnant than in nonpregnant participants (*P* < 0.05), with the magnitude of difference being more significant for lumefantrine than for DHA. *Post hoc* comparisons of PK parameters between males and females, and between pregnant women in their 2nd and 3rd trimesters, revealed no significant differences. The correlation between capillary and venous plasma concentrations taken simultaneously and analyzed using the same assay instrumentation was 0.94 to 1.04 (*r*^2^ = 0.96 for pregnant women and 0.99 for nonpregnant adults), permitting analytical results for either matrix to be interpreted similarly (our unpublished data).

**TABLE 2:** Noncompartmental analysis of artemether, DHA, and lumefantrine PK parameters in pregnant and nonpregnant adults with uncomplicated P. falciparum malaria

PK parameter^*a*^	Pregnant women (*n* = 30)	Nonpregnant adults (*n* = 30)	*P* value^*b*^
Artemether			
Total dose (range) (mg/kg of body wt)	8.1 (5.9, 10.8)	8.6 (7.0, 12.6)	0.03
*T*_max_ (h)	2.0 (1.0, 2.0)	2.0 (1.0, 2.0)	0.52
*C*_max_ (ng/ml)	38.3 (21.2, 71.5)	22.6 (11.3, 44.8)	0.09
AUC_0–8_ (h · ng/ml)	117.8 (56.9, 177.4)^*c*^	81.4 (41.7, 119.0)	0.20
AUC_0–24_ (h · ng/ml)	148.4 (70.7, 221)^*c*^	112.5 (61.2, 170.9)	0.39
*t*_1/2_ (h)	4.6 (2.1, 6.9)	6.0 (3.0, 8.3)	0.13
Dihydroartemisinin			
*T*_max_ (h)	2.0 (2.0, 3.0)	2.0 (2.0, 3.0)	0.91
*C*_max_ (ng/ml)	73.9 (56.4, 106)	70.9 (56.7, 87.7)	0.65
AUC_0–8_ (h · ng/ml)	196.5 (148.0, 261.1)	199.5 (171.1, 259.7)	0.67
*t*_1/2_ (h)	1.3 (1.1, 1.5)	1.5 (1.3, 1.8)	0.01
Lumefantrine			
Total dose (range) (mg/kg)	48.5 (35.5, 64.7)	51.7 (42.1, 75.8)	0.03
*T*_max_ (h)	8.0 (0.6, 8.0)	5.9 (3.0, 8.0)	0.54
*C*_max_ (ng/ml)	6.5 (4.1, 12.4)	5.0 (4.0, 7.1)	0.19
AUC_0–∞_ (h · ng/ml)	318.5 (181.0, 474.0)	266.7 (193.7, 339.8)	0.38
*T*_max_ (h)	106.4 (48.8, 139.3)	128.8 (68.6, 146.6)	0.04
Day 7 concn (ng/ml)^*d*^	409.0 (231.0, 617.0)	383.5 (251.5, 546.0)	0.83
Day 14 concn (ng/ml)^*e*^	138.0 (72.1, 210)	155 (101, 225)	0.39

a All data represent PK parameters following the last (6th) dose of AL. Values are reported as the median (interquartile range), unless otherwise noted. *T*_max_, time to reach maximal plasma concentration; *C*_max_, maximal observed concentration after last dose; AUC_0–∞_, area under the concentration-time curve from time zero to infinity; *t*_1/2_, terminal elimination half-life.

b Wilcoxon rank sum test.

c *n* = 29.

d Sampling done on day 8 due to spacing of doses, as described in Materials and Methods.

e *n* = 26 for pregnant adults and *n* = 28 for nonpregnant adults.

### Adverse events and treatment outcomes.

No significant clinical or laboratory adverse events were observed. Three participants had grade 3 thrombocytopenia on day 0, and all resolved. One nonpregnant adult developed grade 3 neutropenia on day 14, which was attributed to direct effects of malaria and resolved on day 28, with no complications. Overall, 57 (95%) and 53 (88%) of 60 participants achieved PCR-unadjusted ACPR on days 28 and 42, respectively. At 28 days, three pregnant women had recurrent malaria, with one LCF and two LPFs. By day 42, 5 (17%) pregnant (2 LCFs and 3 LPFs) and 2 (7%) nonpregnant (both LCFs) participants had recurrent malaria. All recurrent infections were detected between days 28 and 42 (gestational age, 24 to 37 weeks), and PCR-based genotyping at six loci confirmed that all infections were attributable to new infections rather than recrudescence.

An exploratory analysis of PK-outcome associations was conducted. Using a lumefantrine level of 280 ng/ml on day 7 as a target, 12 pregnant women (40%) and 9 nonpregnant adults (30%) were below this threshold concentration. In pregnant women, the median (interquartile range [IQR]) day 7 lumefantrine concentration in those experiencing recurrent infection (*n* = 5) was 231 ng/ml (218, 234 ng/ml) compared to 421 ng/ml (247, 651 ng/ml) in those with ACPR (*n* = 25) (*P* = 0.08). Four of 5 pregnant women experiencing recurrent malaria had a day 7 lumefantrine level of <280 ng/ml. In nonpregnant adults, the median day 7 lumefantrine levels were 367 ng/ml (IQR, 245, 567 ng/ml) in those with ACPR and 385 and 440 ng/ml in the 2 nonpregnant adults experiencing recurrent malaria. In a comparison of the total dose of lumefantrine (in milligrams per kilogram of body weight) administered over the 3-day period in those adults with reinfection (*n* = 7) to those with ACPR (*n* = 53) (both pregnant and nonpregnant), adults experiencing reinfection had a significantly lower milligrams-per-kilogram dose than those who remained aparasitemic during the 42 days of follow-up (*P* = 0.009). As expected, pregnant women had a significantly lower milligrams-per-kilogram dose than that of nonpregnant adults (*P* = 0.027).

## DISCUSSION

### Pharmacokinetics of artemether and its active metabolite DHA.

Our study showed no significant difference in the exposures (maximal concentration or AUC) of artemether or DHA between pregnant and nonpregnant adults who were treated with the standard six-dose regimen of AL for acute uncomplicated P. falciparum malaria. This contrasts with earlier publications on the PK of AL in pregnant women, which reported reduced exposure of both artemether and DHA in pregnant women compared to that in nonpregnant populations: two Thai studies compared AL PK in pregnant women to a previously published studies of mostly men (27, 32), and the study in Tanzania provided a direct comparison of pregnant and nonpregnant adults using sparse sampling (34). Although considerable variability in artemether PK exposure is noted in the literature, our results for artemether and DHA during pregnancy are similar to the values reported in southwestern Uganda, suggesting some consistency in artemether exposure during pregnancy (32).

This is the first report of a direct comparison of artemether PK between pregnant and nonpregnant adults using intensive PK sampling. Although exposure in pregnant versus nonpregnant adults of either artemether or DHA was not significantly different, a slight decrease in the terminal half-life of DHA during pregnancy was observed. This may be explained by the known pregnancy-related induction of UGT1A9 and UGT2B7 (19), the enzymes responsible for the final glucuronidation and disposition of DHA (22). An assessment of plasma and urinary DHA and its glucuronide products in human studies is needed to validate published *in vitro* and *in vivo* animal findings.

Our understanding of the clinical implications of any alteration in artemisinin (either artemether or its metabolite DHA) exposure in special populations, such as children and pregnant women, is incomplete. The frequency of blood smear collection in the first few days following treatment (daily) and the minor changes in PK parameters precluded our ability to accurately access the association between the rate of parasite clearance and artemisinin exposure; however, all participants cleared parasitemia by day 2.

### Pharmacokinetics of lumefantrine.

Our intensive PK study that benefited from sensitive and specific LC-MS/MS measurement of artemether and lumefantrine, PK sampling out to 21 days (versus 14 days or less in earlier studies), and our direct comparison of lumefantrine PK in pregnant versus nonpregnant adults provides a robust assessment of the effect of pregnancy alone on lumefantrine PK (27, 34, 36, 45, 46). Our finding of no significant difference in the overall plasma exposure or maximal concentration of lumefantrine between pregnant and nonpregnant adults is consistent with the results of a previous study in southwestern Uganda, which directly compared pregnant to nonpregnant women (35). Conversely, our results contrast with those of two Thai studies that compared lumefantrine PK in pregnancy to historical controls and another Tanzanian study, which directly compared the PK between pregnant and nonpregnant women; all of these studies suggest that lumefantrine exposure is lower in pregnancy. The AUC results from our study are comparable to exposure estimates reported previously for a study in pregnant women in Thailand, which also sampled after the last AL dose (27). Specifically, the median lumefantrine AUC was 252 ng · h/ml, compared with 319 ng · h/ml in the current study, indicating the consistency in exposure of the long-acting partner during pregnancy. A comparison of the AUCs between our study and the southwestern Ugandan study is not possible due to differences in PK sampling schemes, and AUCs were not reported in the Tanzania study (34).

Lumefantrine concentrations on day 7 have been shown to correlate significantly with treatment response and typically provide a correlate to overall drug exposure (50). In our study, the lumefantrine concentrations on day 7 or 14 did not differ between pregnant and nonpregnant adults, contrasting with prior studies that compared day 7 concentrations in pregnant women to either historical controls in Thailand or to a within-study control group in Uganda (27, 32, 35, 37). The median day 7 concentration of 409 ng/ml for pregnant women in our study was similar to the predicted median day 7 concentration of 414 ng/ml for pregnant women studied in Uganda (37). However, for nonpregnant adults, our directly measured median day 7 value of 384 ng/ml was notably lower than the predicted value of 566 ng/ml for this population reported in that same study. The reasons for this difference are unclear. Both men and women comprised our nonpregnant group, while the earlier study included women only; nevertheless, we found no significant difference in exposures between men and nonpregnant women.

We detected a modest but statistically significant difference in the terminal half-life of lumefantrine (106 and 129 h for pregnant and nonpregnant groups, respectively). This finding was consistent with a recent population PK analysis of data from Uganda and the lower exposure seen at later time points in an earlier Thai study (27, 37). A shorter lumefantrine half-life is attributed to increased drug metabolism during pregnancy and a more-rapid decline in concentrations, which may affect clinical response, since exposure to the long-acting partner is predictive of the risk for malaria reinfection or recrudescence (25, 28, 51). A simulation analysis of data from pregnant women in Thailand suggested that a longer duration of dosing may provide a longer duration of protection for pregnant women, a hypothesis requiring validation in the field (52).

### Lumefantrine and malaria treatment responses.

Although the clinical efficacy and safety of the standard six-dose AL regimen remain high for both pregnant and nonpregnant adults in sub-Saharan Africa (53), a reduction in lumefantrine exposure may compromise the two primary roles for this partner drug to (i) augment the role of the artemisinin in the cure of acute infection, and (ii) protect the host from new infection (20). Specifically, concentrations of lumefantrine on day 7 (both 175 and 280 ng/ml) have been used as threshold values to predict the risk of treatment failure (38, 39), and a known major metabolite of lumefantrine, desbutyl-lumefantrine, has been shown to be a strong predictor of treatment outcome (52). A recent study from Tanzania reported that day 7 lumefantrine concentrations were significantly lower in pregnant women with PCR-uncorrected treatment failure than those in patients experiencing treatment success, with 2 of 7 pregnant women found to have PCR-confirmed recrudescence (34). Fortunately, in our study, based on PCR genotype-corrected results, all recurrent infections were due to new infections. In addition, although pregnant women had significantly higher parasite densities at the time of diagnosis, AL was equally efficacious at treating the acute infection in the two study groups.

However, when evaluating the link between day 7 concentrations and the risk for recurrent parasitemia, we found lower median day 7 concentrations in pregnant women with recurrent parasitemia (231 ng/ml) than in those with ACPR (431 ng/ml), with 4 of 5 of the pregnant women exhibiting levels of <280 ng/ml. Other factors that are expected to impact PK-outcome associations are the degree of background immunity in the population, which is lower in pregnancy (54), and malaria transmission intensity and seasonality (entomologic inoculation rate in Tororo, ≥310 infectious bites per year) (55, 56). Although the overall PK of artemether, DHA, and lumefantrine did not differ between pregnant and nonpregnant individuals, the shorter lumefantrine half-life combined with the decrease in immunity in pregnancy may necessitate longer durations of treatment or higher doses of lumefantrine to provide improved posttreatment prophylaxis in pregnant women living in such settings that are endemic for the disease (52).

As per current dosing guidelines, all individuals weighing ≥35 kg are treated with the same 480-mg lumefantrine dose (12). Given the trend toward a lower day 7 exposure in those with recurrent malaria, we looked at whether lower total AL dose based on weight (in milligrams per kilogram) was associated with recurrent malaria. Notably, adults with recurrent parasitemia weighed significantly more than those with ACPR and consequently had a significantly lower total dose of lumefantrine (*P* = 0.009). Thus, given the wide range of weights for adults (38 to 81 kg in our cohort of 60 adults), along with our observation of an association of milligrams per kilogram dosing and recurrent infection, further evaluation of the “one-dose fits all” approach for those weighing >35 kg is warranted. It remains uncertain whether the additional of additional weight bands (for all adults) or an extension of the duration of dosing (for pregnant women only), as suggested by others (27, 33), is critical for optimizing AL treatment.

While this study represents the first direct comparison of artemether and DHA PK in pregnant and nonpregnant adults and is among the most extensive intensive PK studies for lumefantrine in pregnant women, there are a few notable study limitations. We elected to perform a parallel-group intensive PK study design, allowing us to compare malaria-infected pregnant women to a group of malaria-infected nonpregnant adult controls. A parallel design in the context of infection allows the direct study of the impact of pregnancy alone on specific PK parameters, informing dosing guidelines specifically for pregnancy (57). Considering intersubject variability, a sample size of 30 in each group permits the detection of clinically relevant changes in PK due to pregnancy. Although a sequential design would reduce intersubject variability and allow a smaller sample size, enrollment of the same women with malaria during and after pregnancy would not have been feasible. Of note, the PK results for artesunate, using a sequential design in which pregnant women were studied during pregnancy when infected with malaria and again 3 months postpartum in the absence of malaria, suggest that acute malaria had a greater impact than pregnancy on the alteration of artesunate PK. Malaria infection itself may reduce first-pass metabolism, oral bioavailability, or systemic clearance (58, 59). While the parasite density was higher in pregnant individuals, the clinical severities of malaria in our two study groups were similar, precluding the analysis of such an effect. Another limitation, due to a lack of adequate sample volume, was the absence of data on desbutyl-lumefantrine, the primary metabolite of lumefantrine that may have a role in antimalarial efficacy (52, 60). Additionally, the analysis of associations between the PK and treatment outcomes was limited by our small sample size, and there was insufficient power to detect differences for artemether, DHA, and lumefantrine between the 2nd and 3rd trimesters.

In conclusion, our direct AL PK comparison between pregnant and nonpregnant adults indicates that the overall PK exposures to artemether, DHA, and lumefantrine are not significantly different during pregnancy, indicating that an adjustment of AL during pregnancy does not appear to be necessary to achieve similar exposure in nonpregnant adults. However, a difference in the elimination half-life of lumefantrine was noted in pregnant women, which may impact the risk of recurrent malaria following treatment. Larger studies in pregnant women investigating whether a longer duration of AL dosing (such as 5 days) or higher total doses of AL reduce the risk of recurrent malaria in areas that are highly endemic for malaria may be warranted.
